# Development of a Spillover Effect Assessment Tool for Age-Friendly Communities

**DOI:** 10.1093/geroni/igaf122.1695

**Published:** 2025-12-31

**Authors:** Sophia Casale, Caitlin Coyle, Shayna Gleason, Kathy Black

**Affiliations:** University of Massachusetts Boston, Red Bank, New Jersey, United States; University of Massachusetts Boston, Boston, Massachusetts, United States; University of Massachusetts Boston, Boston, Massachusetts, United States; University of South Florida, Tampa, Florida, United States

## Abstract

Efforts to measure the impact of age-friendly initiatives are just beginning to take shape. Knowledge to date suggests that there are unexpected positive outcomes of the age-friendly process that go unreported. Addressing this gap, we present the development of a conceptual framework and corresponding practice assessment tool for identifying, documenting, and disseminating age-friendly ‘spillover’. Our framework was developed through the triangulation of multiple data collection methods. A comprehensive inductive review of the age-friendly literature (n = 213); a representative cross-national sample of progress reports (n = 44); and insights from conversations with local, state, regional, and global stakeholders (n = 9) informed the building of the conceptual model and tool creation. Additionally, global pre-testing of the spillover assessment tool was conducted through interviews and feedback questionnaires from an internationally representative sample of age-friendly community country affiliates (n = 23) and additional community-level leaders. Results from this research suggest that although the necessary elements of spillover are alluded to, the full realization of the spillover process as a function of systems change is under-recognized. Pre-testing the tool for assessing spillover in age-friendly community work revealed diverse cultural perspectives on spillover outcomes, but highlighted the ubiquitous nature of spillover as a measurable dimension of impact. This framework and assessment tool development offers cross-national implications for age-friendly practice, systems change policy, and future research and evaluation. The tool demonstrates promise for improving the ability of age-friendly initiatives across international settings with differing resource levels and cultural contexts to assess previously underreported effects that demonstrate systems change.

